# Integrated problem-based learning in the neuroscience curriculum – the SUNY Downstate experience

**DOI:** 10.1186/1472-6920-6-47

**Published:** 2006-09-18

**Authors:** Brian Trappler

**Affiliations:** 1 SUNY Downstate, Kingsboro Psychiatric Center, 681 Clarkson Avenue, Brooklyn, NY 11203, USA

## Abstract

**Background:**

This paper reports the author's initial experience as Block Director in converting a Conventional Curriculum into a problem-based learning model (PBL) for teaching Psychopathology. As part of a wide initiative in curriculum reform, Psychopathology, which was a six-week course in the second-year medical school curriculum, became integrated into a combined Neuroscience block. The study compares curriculum conversion at State University of New York (SUNY), Downstate, with the experiences at other medical centres that have instituted similar curricula reform.

**Methods:**

Student satisfaction with the Conventional and PBL components of the Neuroscience curriculum was compared using questionnaires and formal discussions between faculty and a body of elected students. The PBL experience in Psychopathology was also compared with that of the rest of the Neuroscience Block, which used large student groups and expert facilitators, while the Psychopathology track was limited to small groups using mentors differing widely in levels of expertise.

**Results:**

Students appeared to indicate a preference toward conventional lectures and large PBL groups using expert facilitators in contrast to small group mentors who were not experts. Small PBL groups with expert mentors in the Psychopathology track were also rated favorably.

**Conclusion:**

The study reviews the advantages and pitfalls of the PBL system when applied to a Neuroscience curriculum on early career development. At SUNY, conversion from a Conventional model to a PBL model diverged from that proposed by Howard S. Barrows where student groups define the learning objectives and problem-solving strategies. In our model, the learning objectives were faculty-driven. The critical issue for the students appeared to be the level of faculty expertise rather than group size. Expert mentors were rated more favorably by students in fulfilling the philosophical objectives of PBL.

The author, by citing the experience at other major Medical Faculties, makes a cautious attempt to address the challenges involved in the conversion of a Psychopathology curriculum into a PBL dominated format.

## Background

Problem-based learning, otherwise known as "PBL," has been incorporated into the curriculum at many medical schools around the world [1]. The main purpose of this method is to help students acquire new information by providing them with a context to apply their knowledge to clinical problems. A further aim of PBL is to provide students with resources in self-directed learning skills that will persist throughout their careers [1,2]. When compared with the conventional curriculum, the PBL method generally increases use of limited resources at medical schools, while debate continues as to its advantage in enhancing learning and test performance [3]. Therefore, the architects of the PBL method have encouraged the creation of different methodologies to assess its effectiveness, which has provoked a broad debate on what medical education is attempting to achieve [1].

The most thorough review to date on the value of PBL was published by Albanese and Mitchell, who conducted a twenty-year meta-analysis of the literature comparing PBL with conventional curricula.

In 1999, the Dean of the Medical School at SUNY undertook an initiative to transform the format of the second-year curriculum away from a conventional didactic model, and move towards one that was PBL-based. This directive was applied to all the courses in the second-year curriculum, which were divided into distinct blocks, most of them from four-to-six weeks.

As Course Director in Psychopathology, the author joined a panel representing a cross-section of Academic Neuroscience Faculty charged with the challenge of transforming the Neuroscience block into a PBL-dominated curriculum. This paper will present the way SUNY Downstate Medical Center implemented the psychopathology segment of the curriculum. It will address the methodologies involved in the implementation of the PBL learning method, student satisfaction ratings, and test performance results.

Psychopathology in a PBL format would no longer exist as a separate entity. Instead, it would become incorporated into a Neuroscience block, consisting also of Neuropathology, Infectious Disease, Microbiology, Psychopharmacology, and Clinical Neurology. This required a splitting off of aspects of the general curriculum to create a "Neuroscience bundle." For instance, the Department of Pharmacology would contribute lectures on neurone signal induction, neuropeptides, anaesthetic and tranquilizing agents towards Neuroscience, while topics such as cardiovascular agents and antimicrobials would be bundled into the Medicine block. Of the seventy hours dedicated to the Neuroscience curriculum, twenty hours consisted of Psychopathology lectures and PBL cases. Nine of the twenty hours were devoted to lectures in Child and Adolescent Psychiatry, Geriatrics, Somatoform Disorders, Sleep and Eating Disorders, Personality Disorders, and Drug and Alcohol abuse. Eleven hours were devoted to PBL modules in psychosis, mood disorders, anxiety disorders, and the dementias.

Prior to this initiative, the twenty-hour independent course of Psychopathology had consisted of three clinical videotapes, each containing a one-hour interview followed by an informal discussion, addressing the three core areas of Psychopathology; namely Psychosis, Mood disorders, and Anxiety Disorders, while fourteen hours had been devoted to lectures covering the rest of the spectrum of psychopathy.

In the new curriculum, the Psychopathology PBL track became "integrated" in that the Neuroscience lecture syllabus was structured to synchronize with the appropriate Psychopathology PBL group. For instance, the lecture on "Neuron Receptor Functions," originally delivered as part of the Psychopharmacology syllabus, would now precede the Schizophrenia workshop. Likewise, the lecture on Tranquilizers and Hypnotics would precede the Anxiety workshop. The Neuropathology of the Dementias would precede the Dementia workshop.

## Methods

The study compares two modalities of implementing PBL. Since the Neuroscience Committee was required to make a rapid transition from a conventional to a PBL curriculum with limited time and resources, each sub-block was permitted to use its own discretion according to each department's resources.

The psychopathology curriculum was organized around four case-based modules, which defined the core psychopathology curriculum. These consisted of the Psychotic Disorders, Mood Disorders, Anxiety Disorders, and the Dementias. These modules were chosen because they were judged by the committee as central to the psychopathology course and because they lent themselves to problem-generated discussions, using a problem-based learning model.

Nine hours were left for other aspects of the curriculum that either required specialty knowledge, not easily acquired by most mentors, or because they were regarded as less essential to the training of physicians (an example of this would be the Personality Disorders). These disorders were maintained in lecture form.

In order to adhere to the principals of small workshop formats, where faculty would function in the capacity of facilitator rather than teacher, the psychopathology portion of the Neuroscience block was divided into twenty small groups of ten students. This involved the sustained participation of twenty faculty members over a six-week block of time. The following criteria distinguished between "expert" and "non-expert" mentors: An expert mentor would usually be a faculty member at an associate or full professor level with the following qualifications:

1. Has several years of graduate teaching experience.

2. Has expertise in conducting group therapy with a solid understanding of group process.

3. In most cases has participated in clinical research.

Non-expert mentors were either junior faculty members or clinicians lacking consistent experience in teaching or clinical research.

In contrast, the remainder of the Neuroscience curriculum divided the PBL classes into eight groups of twenty-five students choosing to limit their PBL modules to eight groups of twenty-five students, thus exposing students to a much more consistent level of faculty expertise. This also functioned as a comparison construct.

A fundamental premise of the PBL method is that problem-solving and self-directed acquisition of knowledge creates a dynamic tension that leads to a more active, gratifying, and effective education [4]. In order to achieve this, each PBL committee was charged with selecting a prototypical case report containing clinical and basic science principles, with a design that would impose a progression of challenges and decisions for the student based on evolving data.

The students were provided reading assignments and case vignettes two weeks prior to the commencement of the Neuroscience block. They also attended an introductory lecture prior to the commencement of the Neuroscience course defining the nature and objectives of a PBL format. During the orientation, students were charged with the following tasks:

1. They would be expected to acquire a core knowledge base via self-learning. An "essential reading list" was provided for this purpose (this was later streamlined after the student feedback session).

2. They were provided with a list of basic science and clinical objectives together with a case vignette on each of four subject modules.

In order to create a problem-based learning paradigm, a committee of experts was set up for each module. Each committee was charged with the mission of: 1) generating a case report, 2) using the case as a springboard for fruitful exploration and discussion, 3) providing questions and references for the students that would encourage self-directed reading, 4) creating a user-friendly manual for the mentors, and 5) generating a set of examination questions that would be based upon students' attendance and participation in the case-based learning module. All mentors received orientation sessions in the content and learning objectives in each of the four Psychopathology modules. For instance, the Depression Module presented a simulated patient who overdosed after a broken romance. He had suffered the loss of his mother in early childhood. He was an "overachiever," and somewhat narcissistic, and friends found him difficult to get close to. The vignette purposefully contained a set of data allowing exploration of various developmental theories explaining his vulnerability to depression from the perspective of 1) Self-psychology. 2) Object relations theory, and 3) Psychodynamic theory. Sufficient DSM-4 Criteria for the diagnosis of a Major Depressive Episode were embedded in the case vignette. The students would be expected to identify them. In addition, the vignette contained some personality qualities with sufficient ambiguity to allow discussion about a possible Axis II diagnosis. Learning tasks also included the monoamine theory of depression.

It should be noted that the PBL model employed at SUNY differed from the "pure" PBL model proposed by Howard S. Barrows, where small student groups:

1. Review the learning needs after reviewing the case.

2. Decide on the best learning resources, such as textbooks, monographs, and journal articles, and then,

3. Return from their self-study as "assumed experts, armed with the knowledge necessary to resolve the simulated patient problem."

4. *The student group *then decides on the clinical hypothesis and problem-solving strategies [5].

A questionnaire was circulated at the end of the entire Neuroscience course, probing levels of student satisfaction with conventional lectures, PBL mentors, handout materials and perception of PBL effectiveness in Psychopathology, and the rest of the Neuroscience curriculum. The questionnaires were completed at the end of the Neuroscience block prior to the final exam with a response rate of 80%.

A questionnaire assigning a score of 8 for "strongly agree," 6 for "somewhat agree," 4 for "somewhat disagree," and 2 for "strongly disagree," was provided to the students at the completion of the Psychopathology and entire Neuroscience block. The questions addressed ten items of the course. Favorability was endorsed as positive for a score of 6 or higher. The ratings were subjected to a chi square analysis to assess statistical significance performing multiple chi-square tests on all ten questionnaire items. The results were dichotomised to address the question of favorability versus non-favorability on the ten items in question.

The Course Directors subsequently had a formal feedback meeting with the elected Student Body of eight students to obtain a more specific and elaborate critique of the new curriculum. All second-year students were encouraged to pass on general comments to their representatives in the student body, in an attempt to upgrade the course. The Student Body reported a high turnout to the post-Neuroscience block feedback session, with strong consensus regarding student experiences. These comments also correlated well with the questionnaires but added qualitative depth.

## Results

Prior to the change of curriculum, students' attendance at lectures ranged from 25% to 30%. Attendance of the informal video sessions ranged from 85% to 90%. Attendance at the lectures in the new curriculum increased to 85% and remained at the 85% to 90% level for the PBL workshops. The enclosed Table 1 summarizes the students' response to the Psychopathology component of the new curriculum and the mean response to the entire Neuroscience curriculum.

The Student Body reported a high turnout to the post-Neuroscience block feedback session, with strong consensus regarding student experiences. These comments also correlated well with the questionnaires but added qualitative depth. From the feedback questionnaire and subsequent in-depth discussion with the Student Body, the following salient points emerged regarding the Psychopathology course:

1. Many students believed that in our haste to convert from a conventional curriculum to a PBL model, faculty had sacrificed too many lectures by placing an overreliance on the PBL workshops as a substitute forum to disseminate a core knowledge base. Unprepared for this method, these students were left floundering and frustrated, having to use their own resources to acquire a core knowledge base. This occurred much more frequently in the small psychopathology groups with junior mentors who struggled to utilize case material to convey didactic knowledge. This is reflected in Questions 4 and 5 of the Table 1, where students in the larger Neuroscience groups with expert mentors rated PBL experiences significantly more favorably. In spite of the possibility that the chi-square analysis may be inflated by multiple chi-square tests, this finding was reinforced by the student feedback body's preference for expert mentors in Neuroscience. Students with negative experiences in the Psychopathology track complained that PBL mentors were unable to integrate case material adequately with didactic handouts. Instead, the core knowledge base was conveyed by esoteric references which students found time-consuming and arduous.

2. Traditional lectures, both in Psychopathology as well as Neuroscience as a whole, were still endorsed as highly favorable by a majority of students, as reflected in Questions 6 and 7 in the Table 1.

3. Many students rated the Psychopathology mentors (who had previously functioned as clinical supervisors) as inadequately trained in the PBL format. Students reported that these mentors would frequently revert defensively to a conventional teaching format, using the clinical case-study manuals provided to the PBL mentors as templates for didactic sessions, neither adequately conveying a critical base of knowledge, nor fulfilling the philosophical objective of the PBL method. In comparison, expert mentors both in Psychopathology as well as in the large Neuroscience groups were able to lead the discussion more effectively and integrate the case-generated discussions into the subject matrix. In both small and large PBL groups, mentors in the discussion process actively engaged students. However, "expert mentors" for students who were either respectively disengaged or overbearing used facilitation or containment techniques more effectively. Student representatives and "floating" faculty observers agreed that this played a critical role in maintaining a safe and open work climate. The importance of experience in group process was also demonstrated in that non-experts were more vulnerable to reverting to didactic techniques under pressure.

4. There was a high favorability rating for Neuroscience PBL groups as reflected in students' response to Question 5. In comparison, only those students in Psychopathology assigned to PBL groups led by senior faculty reported having a gratifying experience, resembling the positive responses elicited in the general Neuroscience track where exclusively senior faculty mentors ran PBL groups.

## Discussion

PBL provides a potentially challenging, more motivating, and enjoyable approach to medical education, and may promote lifelong habits of self-directed learning [1]. PBL is, however, more expensive than conventional curricula, especially in larger medical schools [6]. In the early literature reviews, PBL graduates tended to rate their basic science background weaker than their conventional curriculum counterparts. These results suggest that PBL may not develop in students an effective cognitive foundation [7]. Other studies have indicated that while students favor PBL curricula, they also express dissatisfaction about a lack of a structure or direction [8].

Mc Master students identified a lack of definition of core material as a weakness in student-directed PBL [9]. Neame & Powers, in an article titled "Assisting Students to Learn How to Learn," concluded "It is impractical to suggest that an unstructured, undergraduate medical course be designed in which the onus is entirely upon the student to define and undertake his own program of studies." What these authors recommended was a gradual progression towards independent learning, via a graded reduction of imposed structure [10].

Our PBL model emerged in response to an initiative made by the Dean of the Medical School. A Neuroscience Committee consisting primarily of Neuroscience Faculty Heads was established to construct the PBL-dominated curriculum. This report describes student responses following the first semester of the revised curriculum. Since Psychopathology differed in its implementation to the rest of the Neuroscience block, a comparison was made between two modalities. Our PBL model diverged from the original purist construct, where problems are defined by the participants and evolve in a linear progression through a series of workshops dedicated to a single case. Time restraints in our revised Neuroscience curriculum imposed a limit of sessions per topic, necessitating a structure where problems would be faculty-generated, rather than student-generated.

The salient criticisms by students of our curriculum change were two-fold:

1) Discussion in Psychopathology PBL workshops were not rated as highly as lectures delivered by Senior Faculty. 2) Large PBL groups with expert mentors were rated as superior to small groups using mentors of variable expertise.

As a result of these findings, we subsequently modified our curriculum to precede each PBL group with a didactic lecture delivered by a senior faculty member. However, we continued to use faculty-generated PBL cases.

In the Psychopathology module, favorability ratings of student satisfaction varied greatly between mentors. There was much more consistency in the rest of the Neuroscience block, where groups were larger, and facilitators more qualified. Our results coincided with the findings of Davis, et al. that experienced mentors trended towards directive behaviors, and students positively endorsed that, while junior faculty tended to be more student-centered (possibly because of their lack of knowledge base) [11]. Students described expert-mentors as being more able to identify relevant learning issues and gaps in knowledge while maintaining a constructive climate and adhering to the guidelines of the problem-based learning task within the time scheduled. This affirmed the decision of Block Directors in the Neuroscience Course who exercised greater caution by keeping their group size larger in order to consistently expose students to expert mentors. In contrast, running multiple small PBL workshops required enlisting numerous faculty members with varying knowledge, depth, and teaching expertise. Students identified this as a weakness in the new Psychopathology curriculum. The advantage of small student PBL groups proposed by Howard Barrows appears to work by creating tightly knit student groups who steer, direct, and delegate learning tasks that evolve over many sessions. In contrast, in our revised model, expert mentors who actively focused the learning tasks and used their group process skills to function both as group facilitators and leaders offset the advantage of small groups.

Course directors are cautioned to address the need to allocate sufficient time for faculty development in use of the PBL method before making radical curriculum reform. More recent reviews of the literature such as those by Azer [3], at the Faculty of Medicine at Melbourne; Gude [12], at the University of Oslow; and Iputo and Kwizera [13], in South Africa, credit the introduction of PBL at their Facilities for improving student attitudes and performance, using differing outcome measures. However, Azer, at the Faculty of Medicine at the University of Melbourne, qualifies his observations with a word of caution. "PBL tutors usually feel that it is not that easy to change their teaching style to the PBL format. They are sometimes unsure about their role, or what strategy they might use to facilitate student discussion." He then provides a list of recommended strategies in overcoming the adjustment to a process-driven discussion format [3].

Our definition of "expert mentor", while unusual in that it required skills both in PBL content and process, may have had operational significance: In a revised Psychopathology block criticized as being insufficiently didactic, expert mentors may have been rated higher due to their better flexibility in compensating for didactic course deficiencies while continuing to function as group facilitators.

While our findings still confirm the caution expressed by Albanese & Mitchell in implementing comprehensive curricula with rapid conversions to PBL [1], the data also adds some constructive findings to the evolving literature on this important subject. Before launching into a PBL dominated curriculum, faculty should appropriate skill training to prospective PBL mentors to allow them to function comfortably using this teaching format.

An optimal framework may be one that captures the benefits of both conventional and PBL components, with the early dominance of conventional teaching and the introduction of PBL, in increasing complexity, commensurate with student development and faculty resources.

## Conclusion

The author reports the methodological challenges in making rapid curriculum reform at SUNY Downstate Medical School in which the Neuroscience course for second-year medical students was converted from a conventional lecture format into a PBL-dominated format. A comparison was made between the entire Neuroscience course and the Psychopathology track. While Psychopathology used small student groups with mentors of varying experience and expertise, the rest of the Neuroscience block conducted large groups confined to senior faculty functioning as expert mentors. Second-year medical students indicated a preference towards large groups with experienced mentors, which enhanced the appreciation of PBL learning.

Medical schools throughout the world have adopted a PBL learning approach in their curriculum. There is a general consensus that PBL engages more student involvement and challenges self-directed learning. Variations in success at different schools are probably impacted by multiple variables, such as culture, prior learning experience, and educational expectations. While senior faculty usually receives high ratings by students, limited resources usually dictate the allocation of multiple PBL tutors, ranging widely in expertise. Bearing this in mind, Block Directors should allocate appropriate time resources to promote skills that help facilitate process problem-based discussions to provide tutors and students with an educational experience that is both effective and gratifying.

## Competing interests

The author(s) declare that they have no competing interests.

## Pre-publication history

The pre-publication history for this paper can be accessed here:



## Figures and Tables

**Table 1:** Student favorability responses to Psychopathology and entire Neuroscience Curriculum

	**Psychopathology Course**	**Entire Neuroscience Course**	**Analysis of Differences Between Groups (df = 1)**	
			
			**X**^**2**^	**p**
1. There were enough lectures.	88/160	94/160	.13	.72
2. There were enough PBL workshops.	138/160	118/160	.87	.35
3. The quality of the handout material at the lectures was adequate.	118/160	120/160	.01	.92
4. The quality of the handout material at the PBL workshops was adequate.	72/160	124/160	8.64	< .003
5. The mentors were competent in running PBL workshops.	84/160	132/160	6.42	< .01
6. The lecturers were competent.	142/160	144/160	.01	.92
7. The lectures well delivered.	142/160	138/160	.03	.86
8. Lectures should precede PBL seminars.	118/160	94/160	1.64	.200
9. Preference for more PBL seminars.	42/160	47/160	.22	.639
10. PBL seminars were effective.	92/160	115/160	1.55	.212
11. Average Exam Scores.	71.6%	77.2%	Nonsignificant	
